# Assessment of the Efficiency of Non-Invasive Diagnostic Imaging Modalities for Detecting Myocardial Ischemia in Patients Suspected of Having Stable Angina

**DOI:** 10.3390/healthcare11010023

**Published:** 2022-12-22

**Authors:** Kunihiro Iwata, Katsuhiko Ogasawara

**Affiliations:** 1 Section of Radiological Technology, Department of Medical Technology, Asahikawa Medical University Hospital, 2-1-1-1 Midorigaoka Higashi, Asahikawa 078-8510, Hokkaido, Japan; 2 Faculty of Health Sciences, Hokkaido University, N12-W5, Kitaku, Sapporo 060-0812, Hokkaido, Japan

**Keywords:** cardiac imaging techniques, myocardial ischemia, efficiency, decision trees, patient care management

## Abstract

This study aimed to assess and compare the efficiency of non-invasive imaging modalities in detecting myocardial ischemia in patients with suspected stable angina as easy-to-understand indices. Our study included 1000 patients with chest pain and possible stable myocardial ischemia. The modalities to be assessed were cardiac magnetic resonance imaging (CMRI), single-photon emission computed tomography, positron emission computed tomography (PET), stress echocardiography, and fractional flow reserve derived from coronary computed tomography angiography (FFRCT). As a simulation study, we assumed that all five imaging modalities were performed on these patients, and a decision tree analysis was conducted. From the results, the following efficiencies were assessed and compared: (1) number of true positive (TP), false positive (FP), false negative (FN), and true negative (TN) test results; (2) positive predictive value (PPV); (3) negative predictive value (NPV); (4) post-test probability; (5) diagnostic accuracy (DA); and (6) number needed to diagnose (NND). In the basic settings (pre-test probability: 30%), PET generated the highest TP (267) and NPV (95%, 95% confidence interval (CI): 93–96%). In contrast, CMRI produced the highest TN (616), PPV (76%, 95% CI: 71–80%), and DA (88%, 95% CI: 86–90%) and the lowest NND (1.33, 95% CI: 1.24–1.47). Although FFRCT generated the highest TP (267) and lowest FN (33), it generated the highest FP (168). In terms of detecting myocardial ischemia, compared with the other modalities, PET and CMRI were more efficient. The results of our study might be helpful for both patients and medical professionals associated with their examination.

## 1. Introduction

Imaging studies in patients who have stable chest pain and no known coronary artery disease (CAD) are useful in confirming the presence or absence of CAD, when interviews and basic tests such as electrocardiography are insufficient, and in obtaining the required information for patient management. Especially in clinical decision making for patients suspected of having stable angina (SA), using a non-invasive imaging modality to assess myocardial perfusion is essential to predict the risk of future adverse cardiac events and consider the use of invasive coronary angiography [1,2]. Treatments and other interventions derived from the results of cardiac imaging studies will reduce the risk of adverse cardiac events for patients and improve their symptoms and quality of life [3].

Currently, numerous cardiac imaging modalities are available for detecting myocardial ischemia associated with ischemia-causing CAD, including coronary computed tomography angiography (CCTA), cardiac magnetic resonance imaging (CMRI), single-photon emission computed tomography (SPECT), positron emission computed tomography (PET), stress echocardiography (SE), and fractional flow reserve derived from CCTA (FFRCT) [4,5]. These methods are widely performed as non-invasive diagnostic imaging modalities, and many researchers have reported on their diagnostic ability [6,7,8,9]. Recent guidelines offered class 1 recommendations for anatomic examination using CCTA for workup and future risk assessment of patients having stable chest pain and no known CAD on the basis of clinical risk assessment [10,11]. However, CCTA generates exclusively morphological information about coronary arteries. If the uncertain functional significance of coronary artery stenosis is detected, an assessment of myocardial perfusion with functional testing is recommended to confirm the presence or absence of myocardial ischemia and assess the risk of future adverse cardiac events [10,11]. In contrast, regarding functional testing, recent guidelines offered class 1 recommendations for stress testing using CMRI, SPECT, PET, and SE to detect myocardial ischemia without CCTA in intermediate-to-high-risk patients [10].

Studies assessing the ability of functional tests to detect myocardial ischemia primarily use sensitivity and specificity as indices that indicate their ability. However, when explaining the characteristics of these tests, patients without prior knowledge may find them difficult to understand, despite being directly informed of the sensitivity and specificity described in the literature. Therefore, clarifying these indices not only as values obtained from the literature but also as ones that are easy to understand is desirable. Additionally, this practice might be beneficial not only for the patient but also for medical professionals associated with their examination to understand the abilities of each test and to perform appropriate diagnostic tests. Therefore, we aimed to calculate, assess, and compare the efficiency of detecting myocardial ischemia in functional tests using non-invasive imaging modalities for diagnosing SA as easy-to-understand indices by simulation.

## 2. Materials and Methods

### 2.1. Study Design

We analyzed 1000 patients with stable chest pain and any of the following clinical conditions [10,11]:

- Intermediate-to-high risk of major CAD events was expected from the results of the initial evaluation.
- The result of CCTA as an additional test was inconclusive.
- Coronary artery stenosis of uncertain functional significance was detected on CCTA.

A simulation analysis was performed on patients who met the criteria to assess the efficiency of non-invasive diagnostic imaging modalities. As a basic setting, we set the pre-test probability (PTP) of CAD to 30% by referring to past reports [10,12].

We used decision tree analysis [13] to calculate the efficiency, assuming that the aforementioned group of patients would undergo the following five types of examinations:

(1) CMRI (rest and stress perfusion MRI).
(2) SPECT.
(3) SE.
(4) FFRCT (recent guidelines offered class 2a recommendations for FFRCT as a sequential or an add-on testing) [10].
(5) PET.

### 2.2. Literature Search

We performed a literature search to collect data for the simulation analysis. We searched for meta-analyses on non-invasive diagnostic imaging modalities for detecting ischemia-causing CAD that used invasive fractional flow reserve (IFFR) as a reference standard, and we investigated the diagnostic performance (sensitivity and specificity) on a patient basis. IFFR is a reference standard for assessing the severity of CAD and important parameters for coronary-revascularization procedures [14,15]. Furthermore, many studies reportedly use IFFR as a reference standard for assessing the diagnostic ability of non-invasive diagnostic imaging methods for detecting myocardial ischemia.

This literature search was performed using the PubMed database to identify meta-analyses published between January 2015 and January 2021. The search items were as follows: (i) “diagnostic accuracy of coronary artery disease” and (ii) “diagnostic performance of coronary artery disease.” From the search results, we extracted the literature that met the previously mentioned criteria. In the case of multiple results, we extracted the top three articles with the highest number of target patients described in the meta-analysis. In contrast, we selected relatively newer articles if the number of target studies included was similar. Subsequently, we conducted a qualitative evaluation of the literature. Referring to the method reported by Chong et al. [16], the contents of each study were evaluated using the Preferred Reporting Items for Systematic Reviews and Meta-Analyses extension for Diagnostic Test Accuracy (PRISMA DTA) checklist [17]. The PRISMA DTA checklist contains 27 items that assess the quality of meta-analyses. We categorized each checklist item of the candidate literature as follows: “sufficiently described,” “insufficiency described,” and “not described.” While one point was assigned to each checklist item that was “sufficiently described,” zero points were assigned to the rest. Moreover, we calculated the total score of each candidate study. We eventually selected the literature with the highest total score for the analysis. In cases of the same scores, relatively newer literature was selected. Following this evaluation, we extracted the sensitivity and specificity from the selected literature and used them for the data analysis.

### 2.3. Definition of Efficiencies for Detecting Myocardial Ischemia

We defined the efficiencies for detecting myocardial ischemia as follows:

(a) The number of true positive (TP), false positive (FP), false negative (FN), and true negative (TN) results per 1000 patients.
(b) Positive predictive value (PPV) = post-test probability (post-TP (for positive results)).
(c) Negative predictive value (NPV).
(d) Post-TP (for negative results) [18].
(e) Diagnostic accuracy (DA).
(f) Number needed to diagnose (NND) [19].

### 2.4. Calculation of Efficiencies

**Figure 1:**
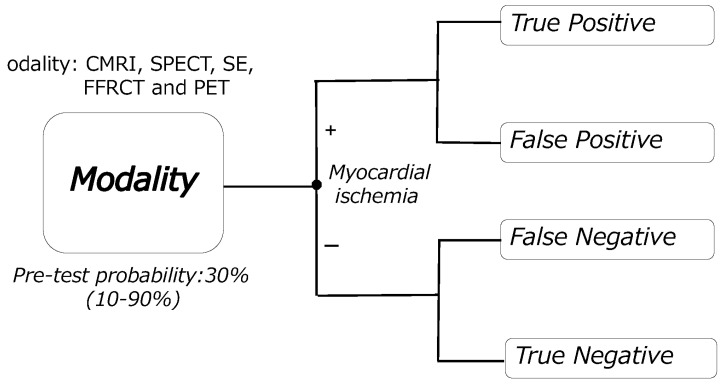
Decision tree model. CMRI: cardiac magnetic resonance imaging, SPECT: single-photon emission computed tomography, SE: stress echocardiography, FFRCT: fractional flow reserve derived from coronary computed tomography angiography, PET: positron emission computed tomography.

In the study’s patient group, we assumed that a workup had been performed to assess the presence of myocardial ischemia. On the basis of the sensitivity and specificity, we conducted a decision analysis using Bayes’ theorem. We calculated the PPV, NPV, and the probability of a positive or negative result from the PTP, sensitivity, and specificity. Moreover, we calculated the probabilities of finally arriving at the endpoint of each branch of the decision tree (Figure 1). Each probability was used to calculate the TP, FP, FN, and TN per 1000 patients. NND was calculated simultaneously, and it represents the required number of patients to be tested to correctly detect the disease in one patient [19]. For the calculation of efficiencies, we used the method published by Hsu et al. [20] to calculate the number of people. The efficiencies were calculated and compared for each imaging modality. Table 1 summarizes the method used to calculate each efficiency. We simultaneously calculated the 95% confidence interval (95% CI) as the point estimates from items PPV to NND described above. We eventually compared the efficiencies of the estimated imaging modalities.

### 2.5. Sensitivity Analyses

The PTP was set at 30% in the basic analysis settings. However, as indicated in the guidelines, the PTP of CAD depends on background factors of the patients, such as sex, age, and symptoms in individual patients [10,11]. Additionally, reports have suggested that performing further downstream testing is appropriate in patients with a pre-test probability >15% [21]. Therefore, we conducted sensitivity analyses to assess the efficiencies, considering the uncertainties associated with the hypothesis-based analysis. The PTP was changed from 10% to 90% (including 15%), centering on an intermediate PTP at which diagnostic tests are considered useful for detecting myocardial ischemia caused by CAD [11]. Changes in the efficiencies in each imaging modality were evaluated and compared. The efficiencies targeted for the sensitivity analyses were limited to those that varied with changes in the PTP. Their post-TPs were calculated using the various PTPs and each efficiency.

### 2.6. Statistical Analysis

The decision analysis and calculations of each efficiency involving 95% CIs were performed using R version 3.6.1 (R Foundation for Statistical Computing, Vienna, Austria, package: epiR) and Microsoft Excel for Mac 2021 Ver.16.56 (Microsoft Corp., Redmond, WA, USA).

## 3. Results

### 3.1. Selected Literature

We extracted nine, six, two, four, and twelve articles on CMRI [6,7,22,23,24,25,26,27,28], SPECT [7,23,24,25,27,28], PET [7,25,28], SE [7,23,27,28], and FFRCT [7,8,9,14,23,27,29,30,31,32,33,34], respectively, for the initial selection (Table 2). Among them, the studies by Pontone et al. (2020) [23] regarding CMRI and SE, Knuuti et al. (2018) [25] regarding SPECT and PET, and Celeng et al. (2019) [31] regarding FFRCT met the inclusion criteria. Therefore, the sensitivity and specificity published in these articles were used for the analysis.

### 3.2. Efficiencies at the Basic Settings

Table 3 summarizes the efficiencies at the basic settings. The order of the calculated number of TP, FP, FN, and TN test results was PET (267) = FFRCT > CMRI > SPECT > SE (192), FFRCT (168) > SPECT > SE > PET > CMRI (84), SE (108) > SPECT > CMRI > PET = FFRCT (33), and CMRI (616) >PET > SE > SPECT > FFRCT (532), respectively. While CMRI had the highest PPV (76%), FFRCT had the lowest (61%). In contrast, PET had the highest NPV (95%) and lowest SE (84%). The post-TP (negative result) of SE was the highest (15.5%), whereas that of PET was the lowest (5.3%). CMRI had the highest DA (88%) and the lowest SE (78%). NND ranged from 1.33 (CMRI) to 2.08 (SE).

### 3.3. Changes in Efficiencies in the Sensitivity Analyses

**Figure 2:**
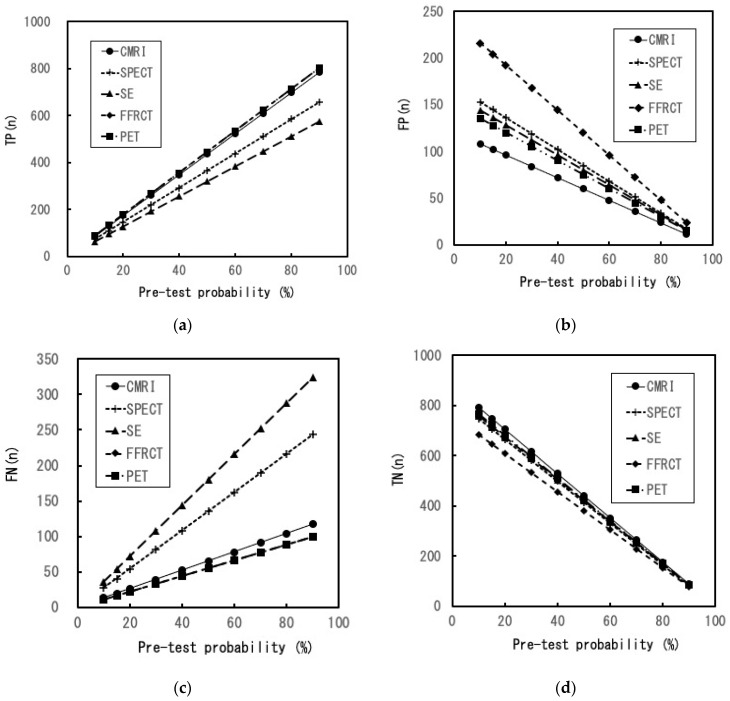
Sensitivity analysis (1). (**a**) Changes in the number of TP results with various pre-test probabilities of CAD. The number of TP PET and FFRCT results is equivalent; (**b**) changes in the number of FP results with various pre-test probabilities of CAD; (**c**) changes in the number of FN results with various pre-test probabilities of CAD. The number of FN PET and FFRCT results is equivalent; (**d**) changes in the number of TN results with various pre-test probabilities of CAD. CMRI: cardiac magnetic resonance imaging, SPECT: single-photon emission computed tomography, SE: stress echocardiography, FFRCT: fractional flow reserve derived from coronary computed tomography angiography, PET: positron emission computed tomography, TP: true positive, FP: false positive, FN: false negative, TN: true negative, CAD: coronary artery disease.

**Figure 3:**
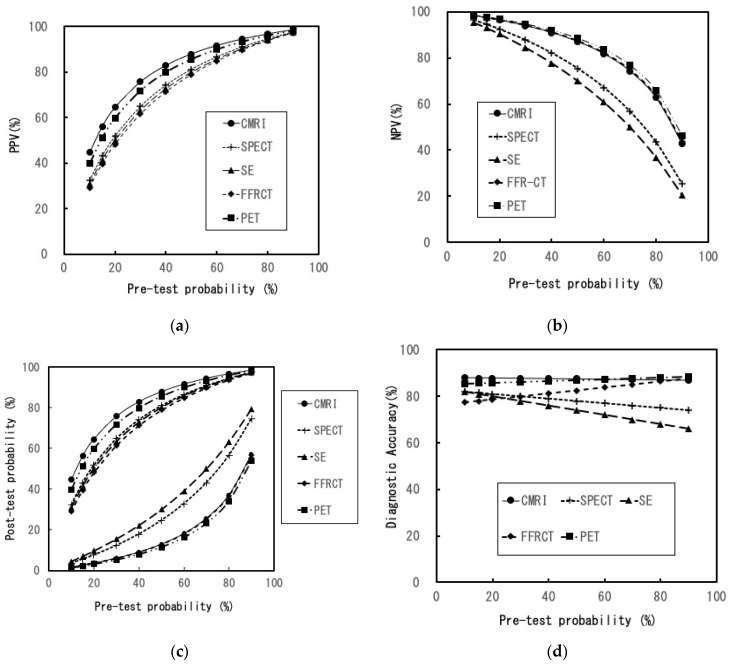
Sensitivity analysis (2). (**a**) Changes in PPV with various pre-test probabilities of CAD; (**b**) changes in NPV with various pre-test probabilities of CAD; (**c**) changes in post-test probability with various pre-test probabilities of CAD, Upper: post-test probability (for positive result), Under: post-test probability (for negative result); (**d**) changes in the diagnostic accuracy with various pre-test probabilities of CAD; CMRI: cardiac magnetic resonance imaging, SPECT: single-photon emission computed tomography, SE: stress echocardiography, FFRCT: fractional flow reserve derived from coronary computed tomography angiography, PET: positron emission computed tomography, CAD: coronary artery disease, PPV: positive predictive value, NPV: negative predictive value.

Figure 2 and Figure 3 depict changes in efficiencies at various PTPs in the sensitivity analyses. In all PTPs, the estimates of TPs for PET and FFRCT were the highest with no change in the order of the other modalities. In addition, FPs, FNs, and TNs were the highest for FFRCT, SE, and CMRI, respectively, with no change in the order of the five modalities. The estimates in CMRI in PPV were the highest in all PTPs, with no change in the order of the modalities. Moreover, PET was the highest in NPV in all PTPs, with no change in the order of the modalities. In the post-TP (negative results), SE was the highest in all PTPs, with no change in the order of the modalities. Furthermore, the estimates in CMRI were nearly constant in DA. With an increase in the PTP, the estimates in DA for PET and FFRCT were increased (up to 4% and 11%, respectively) and for SPECT and SE were decreased (up to 8% and 16%, respectively).

## 4. Discussion

We evaluated and compared the efficiencies of five non-invasive diagnostic imaging modalities for the detection of myocardial ischemia in patients suspected of having SA. Our findings that could be useful to patients were as follows:

Among the five types of modalities in the basic settings (PTP: 30%),

- The maximum and minimum probabilities of a positive test result and having actual ischemia were 76% (CMRI) and 61% (FFRCT), respectively.
- The maximum and minimum probabilities of a negative test result and having no actual ischemia were 95% (PET) and 84% (SE), respectively.
- Despite a negative test result, the minimum and maximum probabilities of existing actual ischemia were 5.3% (PET) and 15.5% (SE), respectively.
- PET generated the best TP and NPV and the least FN among the five imaging modalities.
- CMRI generated the best DA, PPV, and TN and the least FP among the five imaging modalities.
- FFRCT generated the best TP and the least FN among the five imaging modalities but produced more FP results than did the rest.

The DA and NND of PET and CMRI were nearly similar in the basic settings. Regarding the diagnostic ability of SA, it is conceivable that both modalities are roughly equivalent and superior to the other modalities. A detailed evaluation of the calculated efficiency reveals that PET and FFRCT are considered best for patients or physicians who focus on accurate detection and fewer missed diagnoses of myocardial ischemia. The TPs and FNs in FFRCT were equivalent to those in PET. Therefore, when CCTA results are inadequate, adding FFRCT to CCTA might be appropriate, especially if detecting myocardial ischemia is a priority. However, there are some considerations to make when adding FFRCT to CCTA. The number of FPs in FFRCT was the highest among the five modalities owing to the lowest specificity of FFRCT (Figure 2b and Table 2). Especially in the low-intermediate PTP, its FP was substantially higher than that in the other modalities (Figure 2b). The FP results can lead to an inaccurate diagnosis. In addition to unnecessary psychological distress, FP test results in patients with no disease can increase their medical risk because of additional examinations [35].

In routine clinical practice, compared with the other three modalities, SPECT and SE seem to be more accessible. However, NND and post-TP (negative result) of SPECT and SE are higher than those of the other modalities (Table 3). In particular, when the PTP rises, attention must be paid to the marked decrease in DA and the marked increase in FN (Figure 2c and Figure 3d). In contrast, CMRI may be the optimal choice when the patient or physician focuses on higher DA, PPV, and lower FP. Moreover, in all the PTPs, DAs of CMR were high and almost constant, and TNs were the highest among all modalities. From a comprehensive perspective, CMRI is conceivably the most efficient modality for diagnosing SA and the best for its role as a gatekeeper in invasive CAG or coronary revascularization.

Among the non-invasive diagnostic imaging modalities, researchers have primarily conducted studies to evaluate the efficiency of detecting myocardial ischemia or diagnosing SA by economic analysis, such as cost-effectiveness analysis and cost-utility analysis [1,36,37,38]. However, the interpretation of the indicators of efficiency obtained from the results, such as cost-effectiveness ratio and cost–utility ratio, requires a certain degree of specialized knowledge. Therefore, in addition to sensitivity and specificity, patients might find it difficult to understand these indicators, despite being presented directly with the information. To our knowledge, this is the first study that used currently available evidence to assess the efficiency of each modality to detect myocardial ischemia by simulation. We could elucidate the number of TP, FN, FP, and TN per 1000 patients as efficiencies. In addition, by comparing them, we could elucidate the difference in efficiency as a specific index. Similarly, the efficiency of indices, such as PPV, NPV, DA, and post-TPs, was also elucidated and compared. These calculations require setting the PTP. In this study, we were able to assess efficiencies at different PTPs using sensitivity analyses. Thus, our results would conceivably help patients to understand the ability of each examination and undergo the appropriate one. Apart from sensitivity and specificity, the aforementioned indices would be useful not only for patients but also for physicians and other medical professionals associated with the examination. Additionally, physicians can easily understand indicators using NND compared with standard expressions, such as sensitivity and specificity [19]. Furthermore, the physician can also determine the degree of inaccurate diagnosis of SA in each modality by the percentage and number of people, based on the estimated PTP from the results of the medical interview and the basic tests. Based on the above results, it is conceivable that our results are also useful for physicians in selecting imaging modalities for detailed assessment of myocardial perfusion. Therefore, our findings may contribute to reviewing diagnostic strategies and improving the diagnostic workflow for patients with suspected SA through improved understanding of the strengths and weaknesses of each test from the calculated efficiencies.

Our study had several limitations. First, the diagnostic abilities of each modality used to calculate efficiencies were cited from each meta-analysis selected from the literature. In addition, we could not consider the difference in diagnostic ability on the basis of sex. Because we were unable to obtain data from the same patient population, in comparing efficiencies, we deferred the performance of statistical significance tests and only calculated point estimates and their 95% CIs for each efficiency. Thus, a bias could have been introduced. Second, we defined efficiencies as “indices that are easily understood by patients.” However, our results have not yet been applied in clinical practice with actual patients. Therefore, we failed to verify whether patients can understand the calculated efficiencies. This necessitates further verification by incorporating measures such as considering patient opinions.

In addition to this purpose, the primary purpose of using non-invasive functional imaging modalities is to select patients who are likely to benefit from invasive coronary angiography and revascularization [4,39,40]. Although our results suggest that CMRI and PET have superior efficiency compared with other modalities, considering routine clinical practice, conducting CMRI or PET sequentially following basic testing may be impractical. Additionally, the guideline indicates that the choice of a non-invasive imaging test is influenced by patient characteristics, local expertise, and the ease of access to tests [11]. However, we believe that it is worthwhile to evaluate the abilities and characteristics of non-invasive imaging modalities, including CMRI and PET, not only in terms of their sensitivity and specificity but also in terms of their easy-to-understand efficiencies.

## 5. Conclusions

We calculated, assessed, and compared the efficiency of non-invasive imaging modalities for detecting myocardial ischemia in patients with suspected SA. Compared with other methods, PET and CMRI have superior efficiency. Our results revealed the efficiencies of these modalities using the “easy-to-understand index”; they might be helpful for both patients and medical professionals associated with imaging examinations.

## Figures and Tables

**Table 1:** Calculation method for efficiencies per 1000 patients.

		Reference Standard (Invasive FFR)	
		Myocardial Ischemia (+)	Myocardial Ischemia (−)
Index Test	Positive	TP = Sensitivity × PTP × 1000	FP = (1 − Specificity) × (1 − PTP) × 1000
	Negative	FN = (1 − Sensitivity) × PTP × 1000	TN = Specificity × (1 − PTP) × 1000

PPV = post-test probability (positive test result) = TP/(TP + FP), NPV = TN/(FN + TN), diagnostic accuracy = (TP + TN)/(TP + FP + FN + TN), post-test probability (negative test result) = FN/(FN + TN), NND = 1/(Sensitivity + Specificity − 1), PTP: pre-test probability, PPV: positive predictive value, NPV: negative predictive value, FFR: fractional flow reserve, NND: number needed to diagnose.

**Table 2:** List of candidate literature and their characteristics.

Author (Reference)	Year	Modality	FFR Threshold	No. of Studies	No. of Patients	Sensitivity (95% CI)	Specificity (95% CI)	PRISMA Score
Ullah [22]	2020	CMRI	0.75−0.8	17	1886	0.86 (0.79−0.91)	0.86 (0.82−0.90)	15
**Pontone** [23]	**2020**	**CMRI**	**0.75** **−0.8**	**NA**	**1085**	**0.87 (0.84** **−0.90)**	**0.88 (0.85** **−0.90)**	**18**
Yang [24]	2019	CMRI	0.75−0.8	7	718	0.87 (0.73−0.94)	0.87 (0.82−0.90)	-
Knuuti [25]	2018	CMRI	0.8	5	588	0.89 (0.85−0.92)	0.87 (0.83−0.91)	-
Kiaos [26]	2018	CMRI	0.75−0.8	6	516	0.90 (0.85−0.93)	0.85 (0.80−0.89)	-
Danad [27]	2017	CMRI	0.75−0.8	2	70	0.90 (0.75−0.97)	0.94 (0.79−0.99)	-
Jiang [6]	2016	CMRI	0.75−0.8	12	1041	0.87 (0.83−0.90)	0.87 (0.84−0.90)	-
Dai [7]	2016	CMRI	0.75−0.8	15	1054	0.88 (0.85−0.91)	0.84 (0.79−0.87)	17
Takx [28]	2015	CMRI	0.75−0.8	10	798	0.89 (0.86−0.92)	0.87 (0.83−0.90)	-
Pontone [23]	2020	SPECT	0.75−0.8	NA	682	0.71 (0.66–0.76)	0.79 (0.74–0.83)	-
Yang [24]	2019	SPECT	0.75−0.8	8	842	0.72 (0.52−0.86)	0.79 (0.71−0.85)	17
**Knuuti** [25]	**2018**	**SPECT**	**0.8**	**5**	**740**	**0.73 (0.62–0.82)**	**0.83 (0.71–0.90)**	**18**
Danad [27]	2017	SPECT	0.75−0.8	3	110	0.70 (0.59–0.80)	0.78 (0.68–0.87)	-
Dai [7]	2016	SPECT	0.75−0.8	15	1142	0.78 (0.71–0.84)	0.79 (0.70-.087)	17
Takx [28]	2015	SPECT	0.75−0.8	8	553	0.74 (0.67–0.79)	0.79 (0.74–0.83)	-
**Knuuti** [25]	**2018**	**PET**	**0.8**	**4**	**709**	**0.89 (0.82–0.93)**	**0.85 (0.81–0.88)**	**18**
Dai [7]	2016	PET	0.8	4	609	0.90 (0.8–0.95)	0.84 (0.81–0.90)	17
Takx [28]	2015	PET	0.8	2	224	0.84(0.75–0.91)	0.87 (0.80–0.92)	17
**Pontone** [23]	**2020**	**SE**	**0.75** **−0.8**	**NA**	**361**	**0.64 (0.56–0.71)**	**0.84 (0.78–0.89)**	**18**
Danad [27]	2017	SE	0.75−0.8	2	115	0.77 (0.61–0.88)	0.75 (0.63–0.85)	-
Dai [7]	2016	SE	0.75−0.8	6	359	0.69 (0.57–0.80)	0.77 (0.62–0.87)	17
Takx [28]	2015	SE	0.75	4	177	0.69 (0.56–0.79)	0.84 (0.75–0.90)	17
Pontone [23]	2020	FFRCT	0.8	NA	664	0.90 (0.86–0.94)	0.69 (0.64–0.74)	-
Zhuang [14]	2020	FFRCT	0.8	7	1013	0.89 (0.85–0.92)	0.71 (0.61–0.80)	17
Tang [29]	2019	FFRCT	0.8	17	1418	0.90 (0.86–0.92)	0.78 (0.68–0.86)	14
Hamon [30]	2019	FFRCT	0.8	8	823	0.88 (0.84–0.91)	0.72 (0.68–0.76)	-
**Celeng** [31]	**2019**	**FFRCT**	**0.8**	**10**	**1069**	**0.89 (0.85–0.92)**	**0.76 (0.69–0.82)**	**18**
Danad [27]	2017	FFRCT	0.75	3	609	0.90 (0.85–0.93)	0.71 (0.65–0.75)	-
Ding [9]	2016	FFRCT	0.8	4	662	0.90 (0.86–0.93)	0.73 (0.68–0.77)	-
Dai [7]	2016	FFRCT	0.8	4	662	0.90 (0.85–0.93)	0.75 (0.62–0.85)	-
Panchal [32]	2016	FFRCT	0.8	4	662	0.90 (0.85–0.93)	0.72 (0.67–0.76)	-
Wu [8]	2016	FFRCT	0.8	5	833	0.89 (0.85–0.93)	0.76 (0.64–0.84)	-
Gonzalez [33]	2015	FFRCT	0.8	4	662	0.90 (0.85–0.93)	0.72 (0.67–0.76)	-
Deng [34]	2015	FFRCT	NA	4	662	0.90 (0.85–0.93)	0.72 (0.67–0.76)	-

CI: confidence interval. FFR: fractional flow reserve. NA: not available. CMRI: cardiac magnetic resonance imaging. SPECT: single-photon emission computed tomography. SE: stress echocardiography. FFRCT: fractional flow reserve derived from coronary computed tomography angiography. PET: positron emission computed tomography. CTP: computed tomography perfusion. PRISMA: Preferred Reporting Items for Systematic Reviews and Meta-Analyses.

**Table 3:** A summary of efficiencies at the basic settings (Pre-test probability = 30%).

	CMRI	SPECT	PET	SE	FFRCT
Number of TP (*n*)	261	219	267	192	267
Number of FP (*n*)	84	119	105	112	168
Number of FN (*n*)	39	81	33	108	33
Number of TN (*n*)	616	581	595	588	532
Positive predictive value † (%) (95% CI)	76 (71−80)	65 (59−70)	72 (67−76)	63 (57−69)	61 (57−66)
Negative predictive value (%) (95% CI)	94 (92−96)	88 (85−90)	95 (93−96)	84 (82−87)	94 (92−96)
Post-test probability ‡ (%) (95% CI)	6.0 (4.3−8.1)	12.2 (9.8−15.0)	5.3 (3.6−7.3)	15.5 (12.9−18.4)	5.8 (4.1−8.1)
Diagnostic accuracy (%) (95% CI)	88 (86−90)	80 (77−82)	86 (84−88)	78 (75−81)	80 (77−82)
Number needed to diagnose (95% CI)	1.33 (1.24−1.47)	1.79 (1.57−2.10)	1.35 (1.25−1.49)	2.08 (1.78−2.54)	1.54 (1.40−1.74)

† Positive predictive value = post-test probability (positive result). ‡ Post-test probability (negative result). CI: confidence interval. CMRI: cardiac magnetic resonance imaging, SPECT: single-photon emission computed tomography. PET: positron emission computed tomography, SE: stress echocardiography. FFRCT: fractional flow reserve derived from coronary computed tomography angiography. TP: true positive, FP: false positive, FN: false negative, TN: true negative.

## Data Availability

The data that support the findings of this study are available from the corresponding author upon reasonable request.
